# Targeting BRAF and RAS in Colorectal Cancer

**DOI:** 10.3390/cancers13092201

**Published:** 2021-05-03

**Authors:** Helene Bellio, Jean David Fumet, Francois Ghiringhelli

**Affiliations:** 1 University of Burgundy-Franche Comté, Maison de l’université Esplanade Erasme, 21000 Dijon, France; hbellio@cgfl.fr (H.B.); jdfumet@cgfl.fr (J.D.F.); 2 Department of Medical Oncology, Georges François Leclerc Cancer Center—UNICANCER, 1 rue du Professeur Marion, 21000 Dijon, France; 3 Platform of Transfer in Biological Oncology, Georges François Leclerc Cancer Center—UNICANCER, 1 rue du Professeur Marion, 21000 Dijon, France; 4 UMR INSERM 1231, 7 Boulevard Jeanne d’Arc, 21000 Dijon, France; 5 Genomic and Immunotherapy Medical Institute, Dijon University Hospital, 14 rue Paul Gaffarel, 21000 Dijon, France

**Keywords:** BRAF, KRAS, colorectal cancer, targeted therapy

## Abstract

**Simple Summary:**

In colorectal cancer, mutations of the KRAS and BRAF genes are quite common and can contribute to the activation of cell signaling pathways that lead to cell proliferation and differentiation. These processes promote cancer growth, and in some cases, they may cause cells to develop resistance to certain types of treatment, notably EGFR inhibitors. We summarize recent knowledge regarding the effects of KRAS and BRAF mutations in the setting of colorectal cancer and discuss the new therapies under development.

**Abstract:**

Colorectal cancer (CRC) is still one of the most frequent forms of cancer in the world in terms of incidence. Around 40% of CRC patients carry a mutation of the Kirsten rat sarcoma (KRAS) gene, while 10% have a mutation in the B-Raf proto-oncogene serine/threonine kinase (BRAF) gene. These mutations are responsible for dysregulation of the mitogen-associated protein kinase (MAPK) pathway, leading to the proliferation, differentiation, angiogenesis, and resistance to apoptosis of cells. Activation of the MAPK pathway results in adaptive therapeutic resistance, rendering EGFR inhibitors ineffective. This review aims to highlight the recent findings that have improved our understanding of KRAS and BRAF mutations in colorectal cancer and to describe new targeted therapies, used alone or in combination.

## 1. Introduction

According to GLOBOCAN 2018, colorectal cancer ranks third for mortality, after lung and breast cancer, and fourth in terms of incidence worldwide [1]. About 935,000 people died from CRC in 2020, with almost 2 million new cases [1].

CRC represents a heterogeneous group of diseases that are promoted by both environmental risk factors and various molecular pathways, which influence individual susceptibility to cancer. CRC arises after colonic epithelial cells acquire a series of genetic or epigenetic mutations that confer certain capacities for proliferation and immortality [2]. About 70% of CRCs are sporadic, while 20–30% have a hereditary component, such as Lynch Syndrome and familial adenomatous polyposis (FAP) [3].

Next-generation sequencing technologies have enabled improved transcriptomic and genomic analysis of cancer cells and identified many genes and genetic pathways involved in CRC tumorigenesis. The improved knowledge has made it possible to distinguish different subtypes of CRC that are characterized by specific molecular and morphological alterations. EGFR/MAPK, Wnt, Notch, PI3K, and TGF-β signaling pathways are strongly involved in the molecular mechanisms of CRC tumorigenesis and are implicated in the regulation of various processes, such as cell proliferation, differentiation, angiogenesis, apoptosis, and survival. Crosstalk between these pathways leads to overexpression of cyclins and downregulation of CDK inhibitors, which in turn leads to cell cycle disruption and increased cell proliferation [4]. In particular, one of the most frequently mutated pathways in CRC is the mitogen-associated protein kinase (MAPK) pathway. Activation of this pathway promotes proliferation and resistance to apoptosis in most cancer cell types, and determines the efficiency of and resistance to target therapies in the setting of CRC [5]. This is an emerging field, with new drugs targeting RAS or BRAF under development that may soon change the face of CRC therapy.

We review here the role of the MAPK pathway in the development and progression of CRC, and we describe new therapies targeting the BRAF and KRAS genes.

## 2. Classification of CRC

### 2.1. Anatomic and Genetic Classification

The incidence of CRC varies according to the location of the primary tumor. Foods, nutrients, the gut microbiome, and carcinogenic agents probably act in a specific manner determined by the primary tumor location to promote carcinogenesis [6]. Right-sided tumors (RCC) account for approximately 35% of cases, while left-sided (LCC) and rectal cancer represented 65% of cases. Disease progression and overall survival differ according to the position of the CRC. The clinical presentation is different according to the location: the prevalence of anemia at diagnosis is significantly lower in RCC and the prevalence of intestinal obstruction is higher in LCC [7]. A meta-analysis showed the link between the location of primary CRC and overall survival (OS), considering it as a prognostic factor, and patients with RCC had significantly decreased OS compared to patients with LCC [8]. The location of CRC on the right or left side should be considered in the treatment decision in metastatic and possibly even adjuvant stages. In the PETACC8 study, which included only stage 3 cancers, right-sided tumor location was not associated with disease-free survival (DFS) in the overall population, but was associated with shorter survival after relapse [9], suggesting that the poor prognosis of right-sidedness is strongly related to tumor stage, especially metastatic stage. The difference in prognosis between these tumors may be attributed to various factors. Indeed, the right and left colons have a different embryology, whereby the left colon and rectum derive from the hindgut, while the right colon derives from the embryonic midgut. Due to this different embryogenesis, the vascularization of the proximal and the distal colon differ. Other carcinogenic factors also differ between the sides of the colon, such as glucose metabolism, bacterial populations, or exposure to specific nutrients and bile acids [10].

The MAPK pathway is more frequently mutated in left-sided CRC than in right-sided CRC. Accordingly, some studies have reported a high frequency of BRAF mutations, angiogenesis, and T cell activation in RCC in contrast to LCC, which is more frequently associated with WNT and MYC activation [3]. These results suggest different oncogenic processes between these tumor types, with potential repercussions on treatment options, which could change according to tumor sidedness.

On histological analysis, differences between right- and left-sided tumors have been observed. Features of RCC include sessile serrated adenomas or mucinous adenocarcinomas, while LCC display tubular, villous, and typical adenocarcinomas. From a molecular point of view, two main families of CRC have been described. The first accounts for around 15% of CRCs, with hypermutation and microsatellite instability (MSI) secondary to defective DNA mismatch repair (MMR). This family of CRC presents strong immune T-dependent activation. The MSI phenotype is often associated with specific biological features like BAX, β-catenin, TGF-β, and BRAF mutations [5]. In the case of sporadic CRC, BRAF mutation and MLH1 promotor hypermethylation have been observed. A particular CRC group is represented by CIMP+ (CpG Island Methylator Phenotype) tumors, which are characterized by methylation of nucleotide sequences from the promotor region. The methylation of theses sequences is responsible for a defect in the expression of the corresponding genes, more often associated with BRAF mutations. These tumors are more frequently right-sided and found in women [11].

The second family, accounting for approximately 85% of CRC, comprises non-hypermutated, microsatellite stable (MSS) tumors with chromosomal instability. These tumors have a high frequency of DNA somatic copy number alterations and mutation or loss of function in genes such as APC, RAS, SMAD4, PIK3CA and P53, and a small proportion of BRAF mutations. These tumors are more commonly left-sided and are found in men [5].

### 2.2. Molecular Transcriptomic Classification

CRC is a heterogeneous disease whose transcriptomic characteristics could be used to distinguish different forms of CRC presenting particular biological and prognostic features. A large international collaboration proposed a single molecular classification called consensus molecular subgroups (CMS) based on gene-expression analysis. This consensual project used gene-expression data from six different cohorts and proposed the classification of tumors into four different types [12]. The first group, CMS1 (accounting for around 14% of CRCs), is characterized by a high in situ effector immune response, and is frequently associated with right-sidedness and MSI. The second group, CMS2 (37% of CRCs), also called canonical, is mainly characterized by WNT and MYC signaling activation. Genetically, a high degree of chromosomal instability is noted in CMS2 CRC, with mutations in APC, p53, and RAS, but rarely in BRAF. Furthermore, CMS2 is associated with upregulation of the EGFR signaling pathway, with higher expression of the EGFR-ligands amphiregulin and epiregulin [13]. The third group, CMS3 (13% of CRCs), also called metabolic, shows marked metabolic dysregulation and frequent KRAS and APC mutations. Conversely, MSI and CIMP+ phenotypes are less frequent in CMS3. The last group, CMS4 (23% of CRCs), also called mesenchymal, is rarely hypermutated and MSI. It differs from the other groups by overexpression of genes involved in stromal invasion and neo-angiogenesis, activation of the tissue growth factor (TGF-β) pathway, and epithelial–mesenchymal transition (EMT). There are frequent APC, TP53 and KRAS mutations, but only rare BRAF mutations. These characteristics combine to make CMS4 more chemo-resistant than the other subtypes [12] (Table 1).

It is important to underline the fact that 21% of colorectal cancers do not belong to any subgroup, and that heterogeneity of CMS classification may be observed in metastatic sites [13]. In addition, response to chemotherapies seems to be impacted by molecular CMS classification [14].

### 2.3. Immune and Stromal Classification of CRC

Several studies have underlined the major role of in situ immunosurveillance in determining patient prognosis in CRC. Bindea et al. reported that high T cell infiltration is associated with a decreased risk of tumor dissemination and better survival in localized CRC. They proposed standardization of immune infiltration assessment, using the Immunoscore, an automatic assessment of CD3 and CD8 infiltration [15]. Using this score, tumor-bearing patients can be divided into three categories, namely those at high, intermediate, and low risk of recurrence [16]. Moreover, immune response seems to be important in predicting the response to oxaliplatin-based chemotherapy [17]. Interestingly, there is a strong link between molecular classification and immune classification. MSI tumors frequently display T cell immune infiltration. Tumors of the CMS1 and CMS4 subgroups also have high expression of lymphoid and myeloid signatures, and on histological analysis, exhibit a strong immune and inflammatory content, in contrast to the CMS2 and CMS3 subtypes.

In addition to immune parameters, certain stromal parameter may influence patient prognosis. We recently demonstrated that high stromal content is associated with poor outcome [18]. The stromal area is mainly constituted of cancer-associated fibroblasts. CMS4 tumors present high vascularization and inflammatory tumors with a high density of cancer-associated fibroblasts in their microenvironment, which may be implicated in the poor outcome of this category.

## 3. Role of the MAPK Pathway in the Progression of Colorectal Cancer

### 3.1. MAPK Family

MAPK is a member of the large family of Ser/threonine kinases, which comprise three main subgroups, namely: extracellular signal-regulated kinases (ERK MAPK, RAS/RAF/MEK/ERK), c-Jun N-terminal or stress-activated protein kinases (JNK, SAPK), and MAPK14 (p38).

These pathways control many cellular functions including cell proliferation, migration, differentiation and apoptosis. MAPK pathways are activated by several mechanisms including phosphorylation and dephosphorylation, compartmentalization and the scaffolding of MAPK cascades [19]. Stimulation by growth factors leads to the activation of these pathways, including the JNK pathway and ERK1/ERK2s. One of the most important upstream receptors of the MAPK pathway is the transmembrane protein EGFR, which belongs to the ErbB family of receptor tyrosine kinases [20].

**Figure 1:**
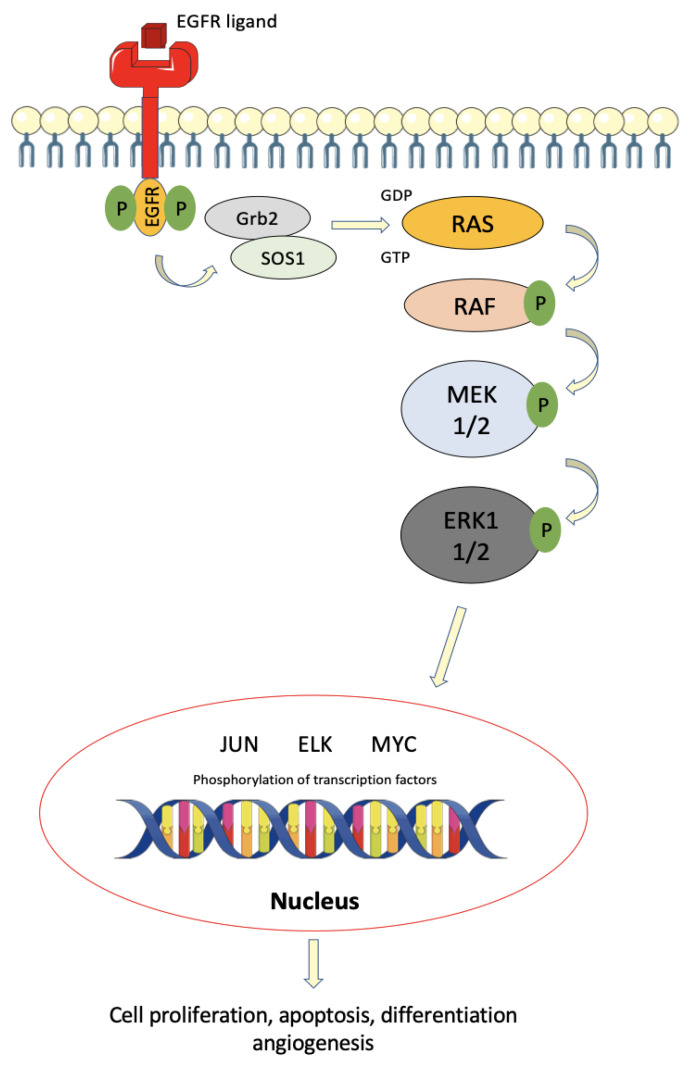
Schematic diagram of the ERK/MAKP signaling pathway in colorectal cancer. Initiation with an extracellular stimulus, EGFR ligand, which binds to and activates the EGFR receptor on the cell membrane. Downstream activation of RAS, RAF, and MEK, in that order, converges in the activation of the ERK1/2 transcription factor activator. This pathway ultimately induces cell proliferation, apoptosis, differentiation, angiogenesis, and metastases.

Following ligand binding, the homo- or heterodimerization of the ErbB receptor leads to induction of auto-phosphorylation of tyrosine residues on this receptor, enabling the release of the Grb2/hSos complex, which in turn activates the RAS and PI3K proteins. RAS alternates between two states, namely an activated state, when bound to GTP, enabling transient interaction of RAS with intracellular effector molecules; and an inactive state, when bound to GDP. Therefore, the activation of RAS is followed by a cascade of phosphorylation of many intracytoplasmic proteins, such as RAF and MEK, which are ultimately responsible for the control of cell proliferation, differentiation, and survival [21] (Figure 1).

The RAS/MAPK pathway is abnormally activated in several cancers, including CRC. Various mechanisms could lead to activation of this pathway in cancer cells, including hyperproduction of ligands such as EGF, TGF-α, amphiregulin, and neuregulin, activating mutations in the Erb receptor or in downstream pathways [22]. This pathway is altered in about 45% of all cancers, mainly due to mutations in BRAF and RAS. In CRC, the most common cause of deregulation of the RAS/MAPK pathway is the presence of activating somatic mutations of genes encoding for the RAS and RAF proteins [23].

### 3.2. MAPK Dysregulation in Colorectal Cancer

The deregulation and activation of this pathway, caused by activation of several proto-oncogenes, is responsible for cell proliferation and survival, and the cell’s ability to invade adjacent tissues [24]. In the last decade, several studies have demonstrated the key role of altered or mutated proteins in this pathway.

First, overexpression of KRAS is present in about 40% of CRCs and is encountered at various frequencies in all CMS, but with a particularly high intensity in CMS3 (68% mutated) [25]. Around 90% of KRAS mutations occur in exon 2 at codons 12 and 13, and 10% in exon 3 or 4, and are an early event in CRC [26]. However, the presence of this mutation alone in colonic cells is not considered sufficient, and a plethora of other alterations is necessary for tumorigenesis. KRAS is not associated with a specific location, but is associated with a well-to-moderately differentiated adenocarcinoma subtype, and the mucinous subtype [27].

Chen et al. reported that in CRC, KRAS mutations were often associated with lymph node involvement and poor prognosis, with a significant decrease in overall survival (OS), in particular those with a KRAS codon 13 mutation, compared with the KRAS wild-type [28]. In a pooled analysis of resected stage III colon cancer patients receiving adjuvant FOLFOX in the PETACC08 and N0147 trials, BRAF or KRAS mutations were found to be independently associated with a shorter time to recurrence and poorer overall survival in patients with MSS status [29].

NRAS mutations are less frequent, and observed in about 4% of CRCs [30]. NRAS mutant tumors are more frequently located on the proximal colon, and are more frequent in older patients [30]. KRAS and NRAS mutant tumors exhibit similar metastasis patterns. Nevertheless, a lower prevalence of mucinous histology and less frequent lung metastases have been reported in patients with NRAS mutation [31].

BRAF mutations are present in about 10–15% of CRCs, with BRAFV600E mutations being the most frequent. BRAF encodes a serine/threonine protein kinase, a downstream effector of the KRAS protein, and BRAF mutation results in constitutive activation of the MAPK signaling pathway. From a molecular viewpoint, BRAF mutations are an early step in the tumorigenesis process, leading to the activation of the MAPK pathway, and they may be a primary event for oncogenesis [32].

BRAF mutations are linked to the CRC location, and have been reported to occur more frequently in right-sided and poorly differentiated tumors, and in females [32,33]. BRAF mutations were also found to be more frequently present in CMS1 (42% mutated) and in MSI tumors [25]. The relationship between BRAF mutations and a high level of microsatellite instability (MSI-H) is also largely demonstrated. BRAF mutations in MSS cancers were associated with a poor prognosis, while the presence of BRAF mutations in MSI-H cancers had no prognostic effect [34]. A previous study investigated the clinical and pathological characteristics of CRCs according to BRAF mutation status, and demonstrated a relation between poor prognosis in mucinous tumors, and BRAF gene mutation, as compared to non-mucinous carcinoma [35]. Thus, mucinous histology appears to be associated with poor response to oxaliplatin- and/or irinotecan-based chemotherapies and with poor OS. Mucinous colorectal carcinomas also have a higher incidence of MSI [36].

BRAF V600E mutation is a marker of tumor aggressiveness, and is associated with poorer prognosis in CRC at the localized or metastatic stage than in BRAF wild-type [37]. In another study, BRAF mutation was significantly associated with more frequent peritoneal and distant lymph node metastases, but less frequent lung metastases, whereas there was no significant difference in the rate of liver metastases between BRAF mutant and wild-type tumors [38]. Mutations in KRAS and BRAF have been described to be virtually (although not completely) mutually exclusive, supporting the proto-oncogene action of both [32,39]. Indeed, it has been suggested that the presence of concomitant KRAS and BRAF mutations may have a synergistic effect on disease progression [40].

Finally, non-V600 BRAF mutations are a very rare and occur in 2% of metastatic CRC patients. These mutations are found in younger patients, mostly males. Tumors are generally well differentiated, are less often located in the right colon, and more frequently harbor concurrent RAS mutations. Survival was shown to be improved compared to metastatic CRC patients with V600E BRAF mutation or RAS wild-type [41].

### 3.3. Involvement in Treatment Response

Survival in metastatic CRC has doubled over the last decade, mainly thanks to new targeted therapies in first-line treatment, such as monoclonal antibodies against vascular endothelial growth factor (VEGF) and against epidermal growth factor (EGFR), and thanks to enhanced usage of chemotherapies, such as irinotecan and oxaliplatin combined in doublet or triplet chemotherapies [42].

Cetuximab, a chimeric IgG1 monoclonal antibody, in combination with chemotherapy, has proven effective compared to chemotherapy alone, in the first and third lines of treatment for metastatic disease [43]. Similarly, panitumumab, a humanized IgG2 monoclonal antibody, has also proved effective with FOLFOX or FOLFIRI compared with chemotherapy alone in first-line therapy, but also as monotherapy in the third line, compared to placebo. Both antibodies are directed against the extracellular ligand-binding domain of the EGFR receptor and block the interaction between EGFR and its ligands [43,44,45].

Several important studies have highlighted a lack of efficacy of cetuximab and panitumumab in metastatic CRC with KRAS mutations, in particular KRAS exon 2 mutations but also KRAS exon 3 and 4 mutations, and similar observations were made for NRAS mutations [46,47,48,49]. Interestingly, while cetuximab appears to be simply inefficacious in patients with RAS mutations, panitumumab appears to be deleterious in RAS-mutated patients, notably when used together with FOLFOX [47]. Accordingly, RAS mutation has become the standard negative biomarker for anti-EGFR therapies. However, many patients with RAS wild-type did not respond to anti-EGFR therapy, and De Roock et al. demonstrated that BRAF and PIK3CA exon 20 mutations were also associated with worse outcome [48]. Despite the binding of EGFR antibodies to EGFR with high specificity, mutations in the KRAS, NRAS, BRAF or PIK3CA genes, which act downstream of EGFR, enable the activation of the intracellular signaling cascade, independently of ligand–receptor interaction. Additional events could explain primary or secondary resistance to anti-EGFR therapies, such as MET amplification, RAS amplification, HER2 overexpression or activating mutation, and mutation of the EGFR extracellular domain, which prevents the anti-EGFR monoclonal antibodies from binding [50].

Other interesting biomarkers have recently been identified, namely AREG (amphiregulin) and EREB (epiregulin), both of which are EGFR ligands. They have been shown to be positive predictive markers for improved outcome with anti-EGFR drugs in two studies, in the setting of treatment with cetuximab [51] and panitumumab [52]. Furthermore, Stahler et al. found a link between mRNA expression of EREG and better outcome in terms of PFS and OS, contrary to AREG [53].

In contrast with lung adenocarcinomas, response to cetuximab or panitumumab in metastatic CRC does not appear to be strongly related to EGFR expression [54]. Nevertheless, some conflicting results suggest that amplification of EGFR could be a biomarker associated with better response to anti-EGFR [55]. However, these data require additional confirmation before clinical usage.

## 4. Targeting BRAF

### 4.1. Different Classes of BRAF

BRAFV600E mutation is the most frequent BRAF mutation, but is not unique, and about 200 different types of BRAF mutant alleles have been identified in all cancers. The most frequent alterations are point mutations, but fusions and in-frame deletions are also observed in some cancers. Indeed, BRAF alterations are classified in three categories based on their biochemical and signaling mechanisms, namely Class I and II mutants, constituting “activators,” and Class III mutants, constituting “amplifiers” [56].

Class I mutants contain only BRAFV600 mutations (E, K, D, R and M). This mutation is the most frequent BRAF alteration, present in about 10% of CRCs, and is capable of signaling as a monomer. These mutations lead to activation of the MAPK signaling pathway, independently of RAS activity, which is suppressed by a negative feedback signal mediated by ERK and results in resistance to anti-EGFR therapy in CRC [56,57].

Class II mutants comprise kinase-activating non-V600 mutations that function as dimers, independently of RAS activity. Similarly, the high ERK activity downstream leads to suppression of RAS activity by a negative feedback signal [58].

**Figure 2:**
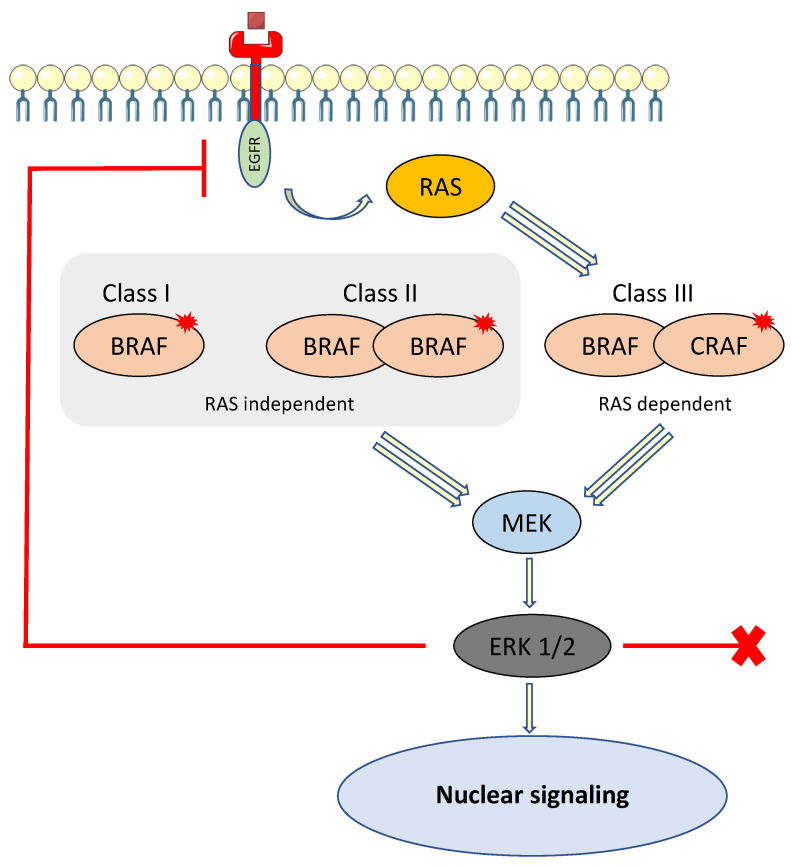
BRAF family and activation of the MAPK pathway.

Class III mutants have impaired BRAF kinase activity and amplify ERK signaling in the presence of activated upstream receptor tyrosine kinases or other coactivators (CRAF). Unlike Class I and II, this class relies on RAS activation to overcome negative feedback from ERK. In CRC with Class III BRAF mutants, RAS is activated by some other mechanisms, such as the high activity of receptor tyrosine kinases (RTKs) [59]. Class III mutants are suspected to have greater sensitivity to EGFR inhibitors and increased overall survival, whereas Class I or II “activators” are classically thought to be intrinsically resistant to EGFR inhibitors [56]. Patients harboring a non-V600 BRAF mutation were shown to have better prognosis compared to V600E BRAF mutation in a retrospective multicenter, cohort study [60] (Figure 2). In this study, Jones et al. reported a marked difference in survival between patients with V600E BRAF-mutant metastatic CRC and those with non-V600E BRAF-mutant CRC (median OS 11.4 vs. 60.7 months, respectively) [60].

Class I and III BRAF mutants are RAS independent, capable of signaling as monomers (Class I) or dimers (Class II), and enable activation of MEK and ERK. ERK is responsible for negative feedback activity by the phosphorylation of RAS, making it inactive. Class III BRAF mutant is RAS dependent, heterodimer, and enables activation of MEK and ERK. RAS is not inactivated by feedback activity.

### 4.2. Response to Anti-EGFR Therapy in BRAF Mutant Colorectal Cancer

Currently, EGFR inhibitors are an important part of the treatment of metastatic CRC, but are known to be ineffective in CRC with activating RAS mutations. Consequently, RAS is becoming a predictive biomarker of the response to EGFR inhibitors, e.g., cetuximab and panitumumab, both monoclonal antibodies. Several studies and a meta-analysis have tested the impact of these antibodies targeting EGFR in patients with advanced CRC who have BRAF mutant status. Results show that the presence of BRAF V600E (Class I mutation) is a negative predictor of response to EGFR inhibitors [61,62,63]. The PICCOLO study demonstrated that panitumumab had a detrimental effect on overall survival in patients with BRAF mutations, without specifying the class of mutation [64].

Likewise, EGFR inhibitors seem to be ineffective in suppressing ERK signaling in Class II BRAF-mutant CRC to induce durable regression. However, a subset of patients appears to respond to these monoclonal antibodies, albeit too rarely to be retained for clinical usage as yet [65]. Conversely, patients with CRC with Class III BRAF mutations have shown sustainable response to EGFR antibodies with first-line chemotherapy. These results demonstrate the main role of EGFR, and patients with CRC with Class III BRAF mutations should be proposed for EGFR inhibitors therapies [65].

### 4.3. Effectiveness of Anti-BRAF Therapy

Three BRAF V600E inhibitors are available for clinical use, namely: vemurafenib, dabrafenib and encorafenib. Since 2010, vemurafenib has proven its efficacy in BRAF mutant metastatic melanoma. Several studies have tested this anti-BRAF therapy in the setting of CRC, but in contrast to the results observed in BRAF V600E melanoma, vemurafenib as a single therapy did not demonstrate any clinically relevant activity in BRAF mutant metastatic CRC [66]. Hyman et al. carried out a phase 2 “basket” clinical trial in order to study the clinical efficacy of single BRAF therapy in multiple types of non-melanoma BRAF mutant cancer. Clinical activity was observed in non-small cell lung cancer (objective response rate 42%), but not in CRCs [67]. These data show that BRAF V600 mutant cancer responds differently to BRAF-targeted therapy depending on the tumor type.

Indeed, the mechanism of vemurafenib resistance in BRAF mutant CRC was studied in cell line models that harbor BRAF V600 mutations. In contrast to melanoma cell lines, vemurafenib therapy failed to permanently sustain reduction of phospho-ERK (P-ERK) expression in CRC cell lines, and after just 24 h, higher expression of P-ERK was observed, mediated by the EGFR-mediated activation of RAS and CRAF, leading to reactivation of the MAPK pathway [68]. Further results in this study showed in vitro and in vivo that the combination of vemurafenib and erlotinib (an EGFR inhibitor) leads to a dramatic tumor response, and an improved inhibition of tumor cell proliferation [68]. This mechanism explains the lower efficacy of BRAF-targeted therapy in BRAF V600-mutant CRC. The efficacy of BRAF targeting therapy in non-V600 BRAF mutant remains unclear (Table 2).

### 4.4. Combination with BRAF Targeting Therapy

Consequently, it has been hypothesized that targeting EGFR or MEK is required in combination with BRAF inhibitors. Such combinations have been tested with a view to making them the standard of care for BRAF V600 mutant CRC treatment. First, preclinical studies demonstrated the efficacy of BRAF-targeted therapy in combination with anti-EGFR monoclonal antibodies and irinotecan [69]. Corcoran et al. reported in a phase 1/2 study the utility of associating a selective BRAF inhibitor, dabrafenib, with a selective MEK inhibitor, trametinib, with a complete response rate of 2% (duration of response over 36 months) and 56% partial response [70]. However, median PFS was only 3.5 months in BRAF-mutant CRC, while it was approximately 9.5 months in BRAF mutant metastatic melanoma [71]. Indeed, a dual MAPK pathway blockade seems to produce a modest antitumor effect for the treatment of BRAF-mutant CRC.

Similarly, a phase 3 study investigated the combination of encorafenib, a BRAF inhibitor with more prolonged pharmacodynamic activity, and cetuximab, with or without binimetinib, an MEK inhibitor, in patients with BRAF V600E-mutated metastatic CRC after one or two previous lines of therapy [72]. Median OS (the primary end point) was 9.0 months in the triplet therapy group and 5.4 months in the control group, corresponding to a 48% decrease in mortality in the triplet therapy group. The authors reported an objective response rate of 26% with triplet therapy, compared to 20% in the doublet therapy group and only 2% in the control group, with better response when patients were treated after one previous line therapy (objective response rate 34%). Despite the triple MAPK pathway blockade, many patients developed resistance to these therapies. Further studies are required to understand these mechanisms of resistance.

Unlike most kinase inhibitors, BRAF inhibitors inhibit their target in all cells. In contrast, V600E BRAF inhibitors selectively inhibit the mutated form of the protein BRAF V600E in tumors, which acts as monomer, but not the wild-type BRAF, which acts as a dimer in normal tissue. This inability to inhibit dimeric BRAF is the basis of their increased therapeutic index, but it is also responsible for the development of drug resistance, based on negative feedback on MAPK pathway inhibition, resulting in rapid formation of RAF dimers and consequent resistance to RAF inhibitors. In addition, these pan-RAF inhibitors induce the RAF paradoxical activation. In the presence of BRAF inhibitors and activated RAS, BRAF forms a heterodimer with CRAF, which triggers the activation of the MEK/ERK pathway at the origin of pathologies such as squamous cell carcinoma [73].

New BRAF inhibitors that target both monomers and dimers or only dimers are in development. These molecules are poorly effective as monotherapy, likely due their side effects related to their capacity to affect wild-type cells. Some of the newer BRAF inhibitors are able to override this paradoxical activation of RAS and avoid the resulting complications [74]. However, a recent report underlined that a combination of BRAF V600E inhibitors plus a dimer inhibitor in addition to an MEK inhibitor promoted the suppression of tumor growth in BRAF V600E therapy-resistant models [75]. Another report showed in preclinical models that combining a BRAF dimer inhibitor with an MEK inhibitor induced a dramatic therapeutic effect and overcame acquired resistance among cancers with KRAS, NRAS, NF1, BRAF non-V600E, and BRAF V600E mutations. Such therapies isolate MEK in RAF complexes, then reduce MEK/MEK dimerization, and uncouple MEK from ERK in acquired resistant tumor subpopulations [76] (Table 2).

## 5. Targeting RAS

Activating mutations in the KRAS gene are seen in 30–50% of CRCs. Such mutations prevent the use of anti-EGFR targeted therapies. The most common somatic mutations observed in KRAS anomalies in CRC are found in codons 12 and 13. Notably, the four most frequent mutations of all CRCs in terms of incidence are G12D (11%), G13D (7%), G12V (7%), and G12C (4%) [77]. This missense mutation aberrantly activates KRAS and results in activation of downstream signaling pathways [78].

### 5.1. K-RAS G12C Inhibitors

The mutation G12C corresponds to an exchange of glycine by a cysteine at codon 12, which catalyzes the exchange of guanine 5′-diphosphate (GDP) to guanine 5′-triphosphate (GTP) and ultimately potentiates weak guanine 5′-triphosphatase (GTPase) activity. Notably, RAS protein alternates between two different conformational states according to binding to different nucleotides, GDP and GTP. GTP-bound RAS activates downstream effector pathways to promote cell proliferation. Two highly mobile regions designated as switch I and switch II allow this modification of conformation, and enable the binding with effector proteins and the activation of diverse downstream effectors, e.g., CRAF, PI3Kα and RALGDS [79].

Recently, Ostrem et al. discovered two allosteric pockets on the surface of RAS that could be targeted by small molecules, between the switch I and II regions of KRAS (switch I/II pocket) [80]. A KRAS inhibitor named BI-2852 binds with nanomolar affinity to a pocket between switch I and switch II, and targets both the active and inactive forms of KRAS [81]. In cell lines, BI-2852 reduced pERK and pAKT levels in a dose-dependent manner, leading to an antiproliferative effect.

ARS-1620, a direct KRAS (G12C) molecule inhibitor discovered by Janes et al., is a small, potent, selective, and orally bioavailable molecule targeting the switch II pocket. It has demonstrated activity in both in vitro and in vivo models for the first time [82].

The first G12C-specific inhibitors to enter the clinic are sotorasib (AMG-510) and adagrasib (MRTX849). Both molecules make it possible to hold KRAS in the inactive GDP-bound state by binding to the mutant cysteine. Sotorasib is an orally atropoisomeric, potent and selective small molecule that specifically and irreversibly inhibits KRAS (GT12C) by binding to a pocket adjacent to the mutant cysteine, used at a dose of 960 mg daily. In a multicenter phase I/II study evaluating AMG-510 (NCT03600883), an objective response rate of 8% and 46% stable disease were observed in CRC patients, with minor sides effects [83]. However, there appears to be a difference in tumor response between lung and colorectal cancer with sotorasib. Indeed, there are other important oncogenic pathways in colon cancer, such as WNT and EGFR [84,85], which interfere with the efficacy of KRAS (G12C) blockade, and KRAS inhibition alone does not appear to be sufficient to impede the oncogenic process [86].

Adagrasib (MRTX849) is also a potent, oral, covalent, selective KRAS (G12C) inhibitor, used at a dose of 600 mg twice daily. It irreversibly binds to the switch II pocket and stabilizes the inactive GDP-bound form of mutant KRAS. In the multicenter phase I/II study evaluating adagrasib (NCT03785249), partial response was observed in one patient, and stable diseases in three CRC patients, also with minor side effects [87]. No individual genetic alterations, e.g., TP53, SKT11, or CDKN2A, seemed capable of predicting the anti-tumoral activity to adagrasib in the cell lines, unlike other KRAS (G12C) inhibitors [87].

Other KRAS (G12C) inhibitors are currently under clinical development, such as the pioneering ARS-1620, which was initially discontinued due to a lack of potency, but is now being studied again in combination with other therapies [88].

### 5.2. Immunostimulatory Effects of KRAS (G12C) Inhibitors

KRAS mutations have multiple roles in immune response. Firstly, they have been shown to be involved in the downregulation of the major histocompatibility complex (MHC) Class I molecules, resulting in an impaired ability of CD8^+^ cytotoxic T cells to recognize cancer cells. Secondly, they have been described to promote tumor inflammation, with the induction of many inflammatory cytokines, chemokines, and several factors involved in inflammation-induced tumorigenesis [89].

In syngeneic CT26 mice models, AMG-510 was shown to increase recruitment of immune effector cells in the tumor environment, such as dendritic cells and proliferating CD8+ cytotoxic T lymphocytes. A synergy between KRAS-targeting therapies and immune checkpoint blockers (ICBs) seems to emerge, with partial response observed in 90% in the AMG-510 plus ICB group [90]. AMG-510 and other KRAS (G12C) inhibitors may represent a potent combination for future use together with ICB-based immunotherapy.

### 5.3. Inhibitors of RAS–SOS Interactions

The son of sevenless (SOS) protein is a key guanine exchange factor (GEF) that promotes the exchange of GDP for GTP, the active GTP state. Conversely, to return RAS to the inactive GDP state, mediators such as neurofibromin (NF1) or RAS GAPs mediate GTP hydrolysis. Depletion of SOS1 or specific alterations in GEF function have been shown to inhibit proliferation of the tumor cells harboring a KRAS mutation, in contrast to cells harboring KRAS wild-type [91]. Besides, SOS1 seems to be an important node in negative and positive feedback regulations of the KRAS pathway.

BI-3406 is a highly potent and selective SOS1–KRAS interaction inhibitor, with proven in vitro and in vivo efficacy. This molecule is able to reduce RAS-GTP and pERK levels and decrease the growth of the majority of tumor cells harboring a KRAS mutation, including G12D and G12V variants, unlike KRAS (G12C) selective inhibitors. Moreover, synergy was observed when SOS1 was combined with MEK inhibitors, blocking the negative feedback pathway. This resulted in tumor stability in colorectal and pancreatic cancer patient-derived xenograft models [92].

A small molecule is currently being tested in a phase I clinical trial as a single agent, namely BI-1701963 (an SOS1 inhibitor), and in combination with trametinib, a selective MEK inhibitor (NTC04111458) (Table 3).

### 5.4. SHP2 and Other Single-Agent Inhibitors

SHP2 is a widely expressed tyrosine phosphatase that acts upstream of RAS in the signaling pathway in the induction of ERK activity. The main role of SHP2 remains unclear, but there is probably a scaffold protein that binds GRB2 and SOS1 and that is required for full activation of the MAPK pathway [93]. Three SHP2 inhibitors are currently under development. The first is RMC-4630, currently tested in a phase I monotherapy clinical trial and a phase I/II clinical trial with cobimetinib, a MEK inhibitor. Second, JAB-3060 is in a phase I/II clinical trial, and third, TNO155 is in a phase I monotherapy clinical trial and a phase I/II trial in combination with adagrasib, a KRAS (GT12C) selective inhibitor [94].

### 5.5. Adoptive Cell Therapy in KRAS Mutant CRC

Durable complete regression of tumors has been achieved in a small number of patients with metastatic melanoma via adoptive cell therapy using ex vivo expanded tumor-infiltrating lymphocytes [95]. It is postulated that this effect is mediated by T cells specifically targeting neoepitopes. Tran et al. described a major response to adoptive T cell therapy in a patient with metastatic CRC [96]. The patient received a single infusion of an enriched population of CD8+ T cells that were reactive to mutant KRAS (G12D), followed by the administration of five doses of interleukin 2. These data suggest that the development of T cell transfer therapy might be useful to target RAS mutated cancer, but may require a better understanding of the binding of KRAS mutants in the HLA system [96].

## 6. Conclusions

After decades of investigation and a lack of treatment, advances in the knowledge about BRAF and KRAS mutations in CRC have led to the development of novel and specific BRAF and KRAS inhibitors. The final results of several ongoing investigations are eagerly awaited, in particular concerning KRAS (G12C) inhibitors and SOS- or SPH2-targeted therapies. The combination with checkpoint blockers and other immune therapies seems very promising and warrants further exploration. Resistance mechanisms have not yet been fully elucidated and require further investigation in clinical trials.

## Figures and Tables

**Table 1:** Biological characterization and clinical prognosis of the consensus molecular subtypes.

Variation	CMS1 MSI Immune	CMS2 Canonical	CMS3 Metabolic	CMS4 Mesenchymal
%	13	34	12	21
Side	Right	Left		
Prognosis	Worse RFS	Better RFS		Worse OS and RFS
Immune parameters	Diffuse immune infiltrate (TH1, cytotoxic cells)	Epithelial differentiation (WNT, MYC)	Metabolic deregulation	TGF-β angiogenesis Complement inflammatory system
Biological parameters	Hypermethylation MSI status high CIMP	High SCNA	Hypermethylation MSI status low CIMP	High SCNA
KRAS (%)	25	25	68	40
BRAF (%)	42	<1	<10	<10

RFS, relapse-free survival; OS, overall survival; TH1, T helper cells; TGF, transforming growth factor; MSI, microsatellite instability; CIMP, CpG island methylator phenotype; SCNA, somatic copy number alterations.

**Table 2:** BRAF inhibitors in metastatic colorectal cancer.

Variation	Reference	Patients, *n* (Type of Study)	Treatment	ORR (%)	PFS (Months)	OS (Months)
BRAF inhibitor monotherapy	Kopetz [66]	21 Phase II	vemurafenib	5	2.1	7.7
	Hyman [67]	10 Phase II “basket”	vemurafenib	0	4.5	9.3
BRAF inhibitor + MEK inhibitor	Corcoran [68]	43 Phase I/II	dabrafenib + trametinib	12	3.5	-
BRAF inhibitor + EGFR inhibitor	Kopetz BEACON [72]	220 Phase III	encorafenib + cetuximab	20	4.2	8.4
Triplet therapy	Kopetz BEACON [72]	224 Phase III	encorafenib + cetuximab + binimetinib	26	4.3	9.0

ORR, objective response rate; PFS, progression-free survival; OS, overall survival.

**Table 3:** RAS and SOS inhibitors in development in colorectal cancer.

Variation	Reference	Patients, *n* (Type of Study)	Treatment	ORR (%)	PFS (Months)	OS (Months)
RAS inhibitor monotherapy	Hong NCT03600883	42 Phase I	Sotorasib	7.1	4.0	-
Inhibitors of RAS–SOS interactions	Hofmann	preclinical	BI-3406	-	-	-
SOS inhibitor + MEK inhibitor	NTC04111458	Phase I	BI-1701963 + trametinib	-	-	-

ORR, objective response rate; PFS, progression-free survival; OS, overall survival.

## Abbreviations

AKT	protein kinase B (PKB)
APC	adenomatous polyposis coli
BAX	Bcl-2-associated X protein
BRAF	v-raf murine sarcoma viral oncogene homolog B1
CDKN2A	cyclin-dependent kinase inhibitor 2A
CMS	consensus molecular subgroups
CRC	colorectal cancer
CRAF	proto-oncogene serine/threonine-protein kinase
DFS	Disease free survival
DNA	desoxyribonucleic acid
EGFR	epidermal growth factor
ERK	extracellular signal-regulated kinase
FAP	familial adenomatous polyposis
GDP	guanine 5′-triphosphate
GRB2	growth factor receptor-bound protein 2
GTP	guanine 5′-diphosphate
GTPase	guanine 5′-triphosphatase
ICB	immune check points blocker
KRAS	Kristen rat sarcoma
MAPK	mitogen-associated protein kinase
MSI	microsatellite instability
MSI-H	high level of microsatellite instability
MSS	microsatellite stable
MYC	c-myc
OS	overall survival
PFS	progression-free survival
PD-1	programmed cell death 1
PDL-1	programmed death-ligand 1
PIK3CA	phosphatidylinositol-4, 5-biphosphate 3-kinase catalytic subunit alpha
RALGDS	ral guanine nucleotide dissociation stimulator
RAS	rat sarcoma
RTK	receptor tyrosine kinase
SHP2	tyrosine-protein phosphatase non-receptor type 11 (PTPN11)
SMAD4	mothers against decapentaplegic homolog 4
SOS	son of sevenless
STK11	serine/threonine kinase 11
TGF-β	transforming growth factor beta
TP53	tumor protein 53
